# Follicular CD8^+^ T Cells: Origin, Function and Importance during HIV Infection

**DOI:** 10.3389/fimmu.2017.01241

**Published:** 2017-09-29

**Authors:** Federico Perdomo-Celis, Natalia Andrea Taborda, María Teresa Rugeles

**Affiliations:** ^1^Grupo Inmunovirología, Facultad de Medicina, Universidad de Antioquia (UdeA), Medellín, Colombia; ^2^Grupo de Investigaciones Biomédicas Uniremington, Programa de Medicina, Facultad de Ciencias de la Salud, Corporación Universitaria Remington, Medellín, Colombia

**Keywords:** follicular, cytotoxic, CD8, T cell, B cell, C-X-C chemokine receptor type 5

## Abstract

The lymphoid follicle is critical for the development of humoral immune responses. Cell circulation to this site is highly regulated by the differential expression of chemokine receptors. This feature contributes to the establishment of viral reservoirs in lymphoid follicles and the development of some types of malignancies that are able to evade immune surveillance, especially conventional CD8^+^ T cells. Interestingly, a subtype of CD8^+^ T cells located within the lymphoid follicle (follicular CD8^+^ T cells) was recently described; these cells have been proposed to play an important role in viral and tumor control, as well as to modulate humoral and T follicular helper cell responses. In this review, we summarize the knowledge on this novel CD8^+^ T cell population, its origin, function, and potential role in health and disease, in particular, in the context of the infection by the human immunodeficiency virus.

## Introduction

Secondary lymphoid tissues, such as lymph nodes, tonsils, spleen, and mucosa-associated lymphoid tissue, favor the collection of antigens (Ags), providing the environment for the close interaction between B, T, and dendritic cells (DCs), macrophages, and other cell types, contributing to the formation of follicles (1). After an antigenic challenge, B cells migrate into the follicle and proliferate to form germinal centers (GCs), aided by follicular cells that provide the proper stimulation to initiate the processes of affinity maturation and immunoglobulin (Ig) class switch and, ultimately, the development of Ag-specific antibody responses (2). A particular transcriptional and phenotypic profile, characterized mainly by the differential expression of chemokine receptors, is required for cellular migration to the lymphoid follicle. CD4^+^ cells are the main T cell subset inside the lymphoid follicle, known as follicular helper T cells (T_FH_). Through the expression of costimulating receptors and the production of cytokines, this specialized cell type constitutes the main help for B cell differentiation and humoral responses against T-dependent antigens (3). However, the partial absence of cytotoxic cells, and their regulatory subset, allows immune evasion by some viral pathogens or tumor cells and the generation of self-reactive B cells after auto-Ag encounter (4). Interestingly, a subset of CD8^+^ T cells was found to express the follicle homing markers, having the ability to enter these areas (here referred as follicular CD8^+^ T cells) (5). In fact, this subset could play variable roles in the control of viral reservoirs and malignancies, as well as in the stimulation and regulation of B and other follicular cells. Here, we discuss the biology of follicular CD8^+^ T cells, their phenotype, transcriptional profile, and differentiation route, emphasizing on their role in the control of acute and chronic viral infections, focusing on the human immunodeficiency virus (HIV).

## Phenotype and Location of Follicular CD8^+^ T Cells

The discovery of B cell lymphoma 6 protein (Bcl6) as the essential transcription factor in a population of CD4^+^ T cells, preferentially located in the lymphoid follicle, led to the recognition of T_FH_ cells as a unique T helper (Th) subset with a gene-expression profile and functions different to those of Th1 or Th2 cells (6). Due to the differential expression of several chemokine receptors, activation markers, and costimulatory molecules, T_FH_ cells are the most numerous T cells in the lymphoid follicle that provide the required signals such as CD40L and a repertoire of cytokines for stimulating B cells (7). Classically, CD8^+^ T cells had been found to be largely excluded from lymphoid follicles, being restricted preferentially to extra-follicular areas, providing an ideal environment for immune evasion of a number of pathogens and the generation of oncogenic processes (8). However, since the last decade, numerous studies have shown that human, non-human primates, and murine CD8^+^ T cells can migrate to the lymphoid follicle and GCs, having an important role in the maintenance of their architecture, pointing their potential participation in supporting B cells while exerting their classical cytotoxic functions.

The first descriptions of a follicular localization of human CD8^+^ T cells emerged from lymphadenopathy studies, where, through immunocytochemistry staining, high frequencies of CD8^+^ T cells were found in inflamed lymphoid follicles (9, 10). Similarly, CD4^+^ T cells, CD20^+^ B cells and CD23^+^ follicular DCs (_F_DCs) constituted approximately the 50% of the cell types in synovial lymphoid follicles from rheumatoid synovitis patients, while CD8^+^ T cells comprised almost the 20% of the follicle area and their presence distinguished the classical GCs. In addition, these CD8^+^ cells also expressed CD40L, a critical molecule for B cell activation and GCs formation, resembling the phenotype of T_FH_ cells (11). Within follicular T cells, the most frequent T cell receptor (TCR) sequences were from CD8^+^ cells, indicating that in this type of inflammatory condition this population is enriched inside the follicle (12).

Quigley et al. first characterized a population of CD8^+^ T cells infiltrating human tonsil follicles and provided the insights into the phenotype and function of follicular CD8^+^ T cells (5). Similar to circulating CD8^+^ T cells, follicular CD8^+^ T cells are activated by peptides presented in the context of class I major histocompatibility complex (MHC); they also express the CD3 receptor, associated with the TCR complex for signal transduction and do not express the CD4 molecule. Importantly, these cells express high levels of the C-X-C chemokine receptor type 5 (CXCR5), critical for entering the B cell zones in secondary lymphoid organs (13), and do not express the C-C chemokine receptor type 7 (CCR7), which directs them to the T cell zones (14). In agreement with the expression of CXCR5, purified human CXCR5^+^CD8^+^ T cells migrate in response to its ligand, the C-X-C motif ligand 13 (CXCL13) chemokine. In addition, follicular CD8^+^ T cells (or CXCR5^+^CD8^+^ T cells) have high expression of the chemokine receptor CCR5, the costimulatory molecules CD27 and CD28, the activation markers CD69 and CD95, and the memory T cell marker CD45RO; by contrast, they express low levels of the cell adhesion molecule CD62L, the glucuronyl transferase CD57, the interleukin (IL)-7 receptor α chain CD127 and the naïve T cell marker CD45RA isoform (5).

In agreement with the expression of memory T cell and activation markers, CXCR5^+^CD8^+^ T cells express the programmed death (PD)-1 receptor (15–18), which binds to its ligands PD-L1 and PD-L2 and inhibits T cell activation (19). Other exhaustion markers such as the T-cell immunoglobulin and mucin-domain containing (TIM)-3 and CD244 (2B4) (20, 21) are not expressed by CXCR5^+^CD8^+^ T cells. The CXCR5^−^CD8^+^ T cells located in secondary lymphoid organs express higher levels of PD-1 and TIM-3 (15, 17), suggesting that CXCR5^+^CD8^+^ T are less susceptible to exhaustion than their counterparts. However, the permanent exposure to Ag in the lymphoid follicle [required for the generation of CXCR5^+^CD8^+^ T cells (15, 16)] during chronic infections or tumor growth could lead to dysfunctional T cell responses (22). Other surface receptors expressed by CXCR5^+^CD8^+^ T cells include the inducible T cell costimulator (ICOS), critical for B cell-T cell interactions (16, 17), and the apparently inhibitory receptor Killer cell lectin-like receptor G1 (15).

The CXCR5^+^CD8^+^ T cells reach the 25% of CD3^+^ cells in follicles from human and murine lymph nodes and spleen (5, 15, 17, 18); a minor fraction (less than 2% of CD3^+^ cells) of human CXCR5^+^CD8^+^ T cells also circulate in peripheral blood, but have a different phenotype in comparison with those confined in the lymphoid follicles, expressing higher CD57, CD45RA, CD62L, and CD127, and lower CCR5 and CD69, although preserving the lack of CCR7 (5). This observation suggests that these cells could downregulate follicle homing markers, egress from the follicle, and enter to circulation, acquiring a less-activated phenotype and possibly migrating to inflamed tissues. The expression of CD62L could be part of a regulated traffic network of follicular CD8^+^ T cells, as in the case of other lymphocyte populations and DCs (23). A summary of some selected phenotypic markers of follicular CD8^+^ T cells is shown in Table 1; the expression levels of these molecules in T_FH_ and B cells is also shown for comparison.

**Table 1:** Phenotypic markers of follicular cells and their function.

Marker	Expression in follicular cells			Function	Reference
	CD8^+^ T cells	CD4^+^ T cells	B cells		
CD3	+	+	−	Associated with the T cell receptor and required for its signal transduction	(5)
CD8	+	−	−	Co-receptor for major histocompatibility complex (MHC) class I molecules	(5, 15, 17)
CD4	−	+	−	Co-receptor for MHC class II molecules	(5)
C-X-C chemokine receptor type 5 [CXCR5 (CD185)]	+/High	+/High	+/High	Receptor for the chemokine CXCL13. It enables the migration of T cells to B cell zones in secondary lymphoid tissues	(5, 15, 17, 24)
C-C chemokine receptor type 7 [CCR7 (CD197)]	−	−	−	Receptor for the chemokines CCL19 and CCL21. It confines T cells to the T cell zones in secondary lymphoid tissues	(5, 17, 24)
CD45RO	+	+	+	Protein tyrosine phosphatase, isoform of CD45 without the A, B, and C exons. Promotes T cell activation. Marker of memory T cells	(5, 25)
Programmed death (PD)-1 (CD279)	+	+	−	Regulate T cell activation and tolerance mechanisms	(15–17, 26)
Inducible T cell costimulator (ICOS) (CD278)	+	+	−	Costimulator of T cells through the binding to ICOSL in B cells	(16, 17, 27)
CD27	+	+	+	Costimulatory of T and B cells through the binding to CD70	(5, 28, 29)
CD62L	−	−/+	+	Cell adhesion and lymph node homing molecule	(5, 17, 30, 31)

## Transcriptional Profile of CXCR5^+^CD8^+^ T Cells

**Figure 1:**
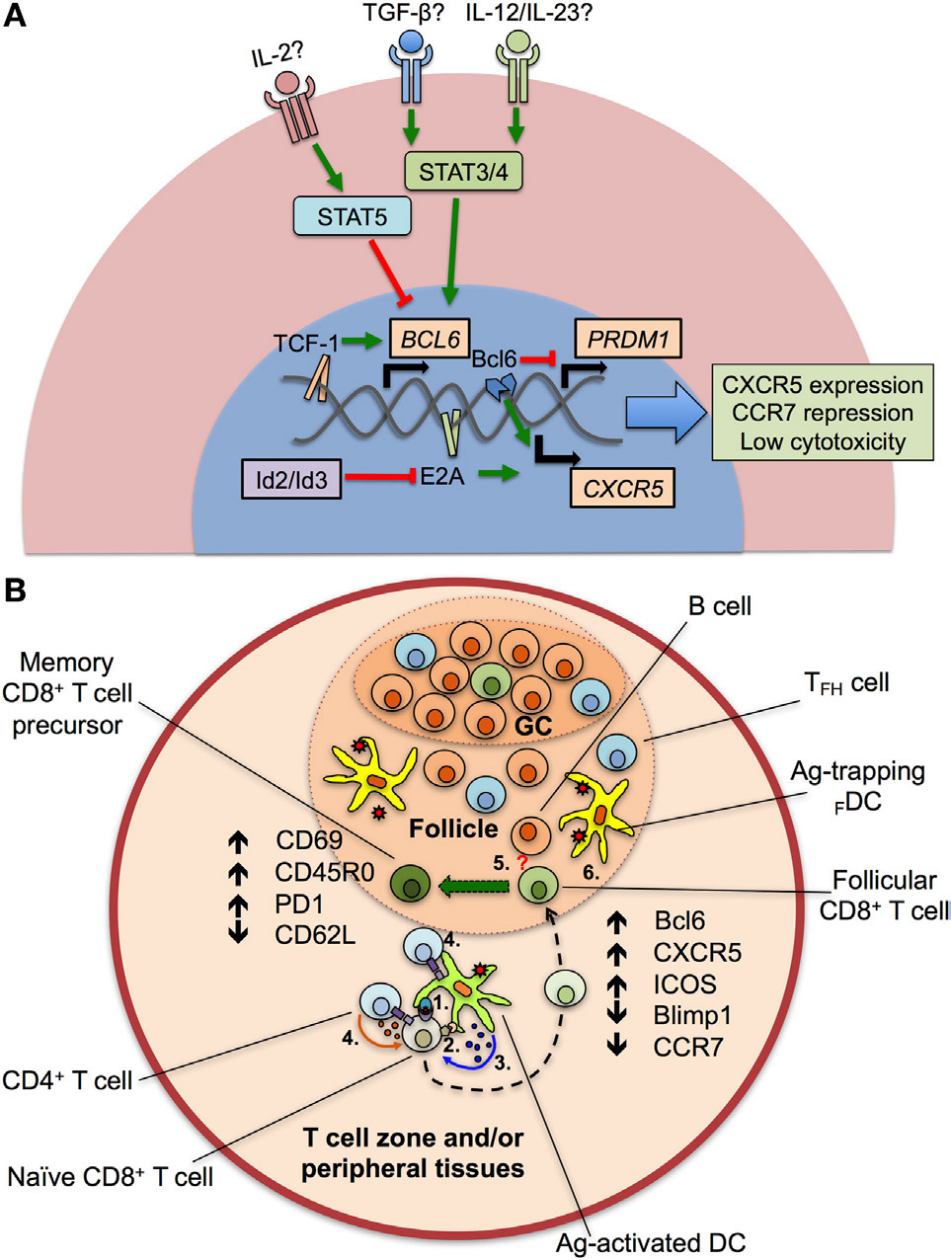
Transcriptional program and differentiation of CXCR5^+^CD8^+^ T cells. **(A)** After the—still to be confirmed—stimulation of transforming growth factor (TGF-β) plus interleukin (IL)-12 or IL-23, the signal transducer and activator of transcription (STAT) 3 and 4 proteins are activated and induce the expression of *BCL6* gene, *via* the binding of the Transcription Factor 1 (TCF-1) to its promoter. TCF-1, together with the B cell lymphoma (Bcl) 6 transcription factor also represses the expression of the *PRDM1* gene, which codifies for the Blimp1 protein. E2A protein, regulated by the inhibitors of differentiation (Id)2 and Id3 proteins, aided by Bcl6, upregulates the chemokine receptor CXCR5, and downregulates CCR7 and cytotoxic activity. Apparently, IL-2, through STAT5 signaling, potently suppresses the expression of *BCL6* gene and the differentiation of CXCR5^+^CD8^+^ T cells. **(B)** After antigen (Ag) processing in T cell zones and/or peripheral tissues, dendritic cells (DCs) present peptides to naïve CD8^+^ T cells *via* class I major histocompatibility complex–T cell receptor interaction (1). Ag-activated DCs also provide costimulatory signals (such as CD80/CD86 binding to CD28) (2) and secrete cytokines that drive the differentiation of CXCR5^+^CD8^+^ T cells (3). The help of CD4^+^ T cells through CD40L–CD40 interaction and cytokine production could be also required (4). Later, differentiating CD8^+^ T cells migrate into the lymphoid follicle and germinal center (GC) and begin to express Bcl6, CXCR5, inducible costimulator (ICOS) and suppress Blimp1 and CCR7. Aided by the potential stimulation of differentiating CD8^+^ T cells by B cells (5, question mark) and Ag persistence (6), CXCR5^+^CD8^+^ T cells fully differentiate and acquire an activated, memory T cell-like phenotype, with high expression of CD69, CD45RO, programmed death (PD)-1, and low CD62L.

When compared with T_FH_, CXCR5^+^CD8^+^ T cells also activate a number of similar transcription factors, driving their differentiation and survival within the lymphoid follicle (Table 2). Nevertheless, this transcription program dictates the reduction of the classical cytotoxic effector functions of CD8^+^ T cells to assure their entry to the follicle. The classical transcription factor of T_FH_ and CXCR5^+^CD8^+^ T cells is Bcl6, critical for the formation of GCs B cells (32); Bcl6 is a transcriptional repressor that belongs to the zinc-finger family of transcription factors. Together with corepressors, Bcl6 targets DNA-damage response genes, inducers of apoptosis and cell cycle arrest, although this regulation has been extensively described in B but not in T cells (33). *Via* the inhibition of the activator protein 1 (AP-1), Bcl6 also represses the expression of *PRDM1* gene (34), which codifies for the transcription factor Blimp1, counterpart of Bcl6 in the differentiation of follicular T cells (35). In fact, which specific genes are differentially modulated by Bcl6 and Blimp1 for the generation of follicular T cells is not fully defined. Blimp1 is a classical anti-proliferative transcription factor, inducer of the secretory machinery and inhibitor of the GC formation (36). Also, in T cells, Blimp1 inhibits the production of IL-2, critical for their proliferation (37). In CD8^+^ T cells, the expression of Blimp1 results in their differentiation to effector and memory subsets. Instead, Blimp1-deficient CD8^+^ T cells generate memory precursor effector cells with low expression of cytotoxic molecules (38). In addition, Bcl6 is upregulated in memory CD8^+^ T cells (39) and suppresses granzyme B expression (40). Thus, Bcl6 and Blimp1 activity reciprocally regulates CD8^+^ T cell differentiation and, due to their expression of Bcl6 and repression of Blimp1, CXCR5^+^CD8^+^ T cells possess follicular helper-like characteristics but potentially decreased cytotoxic functions (17) (Figure 1A), as further discussed below.

**Table 2:** Transcription factors and regulatory proteins driving differentiation of CXCR5^+^CD8^+^ T cells.

Transcription factor or regulatory protein	Expression	Function	Reference
B cell lymphoma 6 protein (Bcl6)	+	Promotes the differentiation of follicular CD4^+^ and CD8^+^ T cells and germinal center (GC) formation	(16, 17, 35)
E protein 2A [E2A or transcription factor 3 (TCF3)]	+	Regulates the expression of surrogate and antigen (Ag) receptor genes; promotes immunoglobulin and T cell receptor (TCR) rearrangements and coordinates cell survival and proliferation with developmental progression in response to TCR signaling	(15, 17, 41)
T cell Factor 1 (TCF-1)	+	Binds to the *Bcl6* promoter and *Prdm1* regulatory regions, activating Bcl6 and repressing Blimp1 expression. Also downregulates genes involved in T cell exhaustion pathways	(17, 42, 43)
Blimp1 [or PR domain zinc-finger protein 1 (PRDM1)]	−	Prevents the differentiation of follicular CD4^+^ and CD8^+^ T cells and GC formation	(16, 17, 35)
Inhibitor of differentiation 2 (Id2 or inhibitor of DNA binding)	−	Binds to, and inhibits the formation of E protein dimers, thus blocking their activity	(15–17, 44)
Inhibitor of differentiation 3 (Id3 or inhibitor of DNA binding)	+		

Interestingly, Blimp1 but not Bcl6 has a binding motif in the *CXCR5* gene, and negatively regulates the CXCR5 expression. The E protein 2A (E2A) also has a binding sequence in the CXCR5 promoter and is, at least in part, the responsible of its expression in CXCR5^+^CD8^+^ T cells, being critical for their differentiation (17). In addition, E2A also binds to the loci of *Tcf7, Bcl6*, *Id2*, and *Id3* [the latter coding the inhibitors of differentiation 2 and 3 (Id2 and Id3)], all of them transcription factors or regulatory proteins in CXCR5^+^CD8^+^ T cells signature. The E protein family is characterized by the presence of a C-terminal basic DNA-binding domain and two helix-loop-helix domains. For full transcriptional activity, these latter domains allow the dimerization of E proteins and DNA binding, event that is prevented by the Id2 and Id3 proteins (44) (Figure 1A). Of note, Id2 likely is the most important regulator of E2A and suppressor of CXCR5^+^CD8^+^ T cells differentiation, as its expression is downregulated in these cells, while Id3 is highly expressed, preferentially in those expressing PD-1 (15, 16, 45).

Another important transcription factor in CXCR5^+^CD8^+^ T cells is the T cell Factor 1 (TCF-1), which is codified by *Tcf7*, and activated *via* the Wnt/β-catenin pathway. In fact, *Tcf7*-deficient mice have a poor differentiation of CXCR5^+^CD8^+^ T cells (16, 17). Among other functions, this factor promotes the formation of memory CD8^+^ T cells and the differentiation of Th2 profile and T_FH_ cells (46–48). In the latter case, TCF-1 binds to the *BCL6* and *PRDM1* regulatory regions and up- and downregulate, respectively, the expression of both transcription factors (42). TCF-1 has been even used as a surrogate marker of CD8^+^ T cells present in secondary lymphoid tissues, distinguishing two populations: a TCF-1^High^TIM-3^Low^ with a gene signature similar to T_FH_ cells and TCF-1^Low^TIM-3^High^, with a transcriptional profile similar to Th1 cells. In addition, TCF-1 is required for sustained viral control during chronic infection (43). Finally, other transcription factors expressed to a lesser degree by CXCR5^+^CD8^+^ T cells include Eomes and T-bet (16), both needed for the generation and maintenance of memory CD8^+^ T cells (49). This latter finding, in addition to the following characteristics suggests that CXCR5^+^CD8^+^ T cells are precursors of memory CD8^+^ T cells: i. the differential expression of Id3 and Id2 proteins (16, 50); ii. the expression of genes associated with fatty acid beta-oxidation; iii. the expression of genes related with self-renewal, progenitors of hematopoietic stem cells and the mammalian target of rapamycin signaling (16).

## Insights into the Differentiation of CXCR5^+^CD8^+^ T Cells

Although the phenotype and transcriptional profile of CXCR5^+^CD8^+^ T cells is partially known, the differentiation factors remain undefined. In the case of T_FH_ cells, a canonical three-step differentiation pathway has been described (51). It initiates with DCs priming of naïve CD4^+^ T cells after an antigenic challenge, where the three classical signals of activation (MHC–TCR binding), survival (costimulatory molecules such as CD80/CD86), and differentiation (cytokines) are provided (52). Similar stimuli, besides of CD4^+^ T cell help *via* CD40L–CD40 interaction and probably IL-2 production, are required by naïve CD8^+^ T cells (53). CD4^+^ T cells committed to differentiate into T_FH_ cells begin to express Bcl6, CXCR5, and ICOS, suppress Th1, Th2, or other profiles (6), and migrate to the lymphoid follicle where they receive signals by other antigen-presenting cells (APCs) such as B cells (e.g., ICOS ligand), inducing effector functions of T_FH_ cells like IL-21 production, a hallmark of this population (54). A remaining question is which cytokines are required for the differentiation of follicular CD4^+^ and CD8^+^ T cells. At this point, there is great divergence between species. In mice, IL-6 plus IL-21 are required for the differentiation of T_FH_ cells (55). By contrast, when human IL-21-expressing CD4^+^ T cells are analyzed, these cytokines are not sufficient to generate T_FH_ cells (56) and, apparently, the optimal conditions for their differentiation require the presence of transforming growth factor (TGF)-β plus IL-12 or IL-23, which act through signal transducer and activator of transcription (STAT) 3 and STAT4 signaling pathways (57). In addition, IL-2 potently inhibits T_FH_ cells differentiation *via* STAT5, inducing Blimp1 expression and Bcl6 repression (58) (Figure 1A). Strikingly, early stimulation with IL-6 induces the production of IL-21 by murine naïve CD8^+^ T cells to levels similar to those of CD4^+^ T cells through a STAT3-dependent mechanism (59), and TGF-β *in vitro* stimulation of CXCR5^−^CD8^+^ T cells from rhesus macaques induces the generation of CXCR5^+^CD8^+^ T cells (45), suggesting that similar differentiation programs are required for mice, non-human primates, and human follicular CD4^+^ and CD8^+^ T cells. Taken together, the available data indicate that, after Ag encounter, DCs, helped by CD4^+^ T cells, prime and provide specific cytokine signals to naïve CD8^+^ T cells that begin to express Bcl6 and CXCR5, among other chemokine receptors, activation and exhaustion markers, migrate to lymphoid follicles where they receive signals from other APCs and antigenic persistence and ultimately differentiate to follicular CD8^+^ T cells with a T effector memory phenotype, most likely constituting the pool of precursors of long-lived memory T cells (Figure 1B).

## Effector Functions of CXCR5^+^CD8^+^ T Cells

CD8^+^ T cells are classically known as cytotoxic cells due to their capacity to eliminate malignant or infected cells (60, 61). In a general scenario, after Ag recognition, preformed and newly synthesized cytotoxic granules in CD8^+^ T cells migrate within the cytosol *via* microtubules translocation and localize in the area in contact with the target cell (62, 63). These granules contain the membrane pore-forming proteins perforin and granulysin, the serine proteases granzymes A-H, the matrix proteoglycan serglycin and cathepsins. After their release, perforin and granulysin polymerize in the membrane of the target cell in a calcium-dependent manner and form pores, resulting in osmotic lysis. In addition, probably mediated by the mannose-6-phosphate receptor, granzyme B enters the target cell, activates caspases, induces the release of cytochrome *C* from the mitochondria and ultimately leads to cell death (64). CD8^+^ T cells also induce apoptosis through Fas ligand–Fas interaction *via* a calcium-independent pathway (61). Non-lytic effector functions of CD8^+^ T cells include the production of IFN-γ, β-chemokines [such as regulated upon activation, normal T-cell expressed and secreted (RANTES), and the macrophage inflammatory protein (MIP)-1α] and the CD8 antiviral factor (CAF) (65). Among other functions, IFN-γ induces the expression of MHC molecules and activation of macrophages and Th1 subset (66), while β-chemokines attract immune cells to inflammatory tissues and, along with CAF, inhibit HIV replication (67, 68).

As previously mentioned, the transcriptional program of CXCR5^+^CD8^+^ T cells drives them to a functional profile different from that of classical CD8^+^ T cells, summarized in Table 3. A comparison between CD44^+^CD62L^−^CXCR5^−^CD8^+^ cells and CD44^+^CD62L^−^CXCR5^+^CD8^+^ cells, both isolated from spleen of P14 mice (with transgenic expression of a LCMV gp33-specific TCR) after 8 days of LCMV Docile strain infection, showed that the latter subpopulation had lower expression of granzymes A and B and perforin, in addition to a lower ability for *ex vivo* elimination of LCMV Ag-pulsed splenocytes (17). Similar findings in the expression of these cytotoxic molecules were obtained at the transcriptional and protein level in CXCR5^+^CD8^+^ cells from mice with LCMV chronic infection (16). Likewise, when the percentage of perforin-negative cells was compared, simian immunodeficiency virus (SIV)-specific CD8^+^ T cells, located in lymphoid follicles from SIV-infected macaques, had a higher frequency than CD8^+^ T cells in extra-follicular areas (18). However, in remarkable contrast, murine CXCR5^+^CD8^+^ cells isolated in late time points after LCMV infection, mixed with Ag-pulsed cells, and transferred into naïve mice had higher *in vivo* killing efficiency than CXCR5^−^CD8^+^ cells. These Ag-specific CXCR5^+^CD8^+^ cells also showed high expression of the degranulation marker CD107 after peptide stimulation (15). In addition, IFN-γ-producing CXCR5^+^CD8^+^ human cells also expressed higher CD107 and perforin than CXCR5^−^CD8^+^ cells in HIV-infected individuals (15). Likewise, in a fraction of follicular SIV-specific CD8^+^ T cells the expression of perforin and granzyme B varied from low to medium in a macaque model of SIV infection (4, 18). Strikingly, even when CXCR5^+^CD8^+^ T cells expressed low cytotoxic molecules, they conserved their proliferation capacity (18) and could decrease the viral load in LCMV and murine herpes virus-infected mice and SIV-infected macaques (15, 17, 18), and the depletion of CD8^+^ T cells led to the increase of SIV-producing follicular and extra-follicular cells (18). In extent, the number of CD8^+^ T cells in follicular compartments inversely correlated with the frequency of SIV RNA^+^ cells in the same areas (4).

**Table 3:** Effector functions and molecules of CXCR5^+^CD8^+^ T cells.

Function/Molecule	Expression/production	Function	Reference
Cytotoxic activity	+/−	Elimination of malignant or infected cells. The capacity of CXCR5^+^CD8^+^ T cells to exert this function depends on the expression of granzymes and perforin	(15, 17)
Granzyme A and B	+/−	Serine proteases that induce cytolysis through caspase-dependent and -independent mechanisms	(17)
Perforin	+/−	Induces the formation of pores in the membrane of target cells for direct cell lysis and for the delivery of the content of cytotoxic granules	(12, 17)
Interferon (IFN)-γ	+/low	Macrophage activation, increased expression of major histocompatibility complex (MHC) molecules and antigen (Ag) processing components, such as tapasin and cathepsins. Induction of immunoglobulin (Ig) class switching and Th1 profile	(15, 17)
Tumor necrosis factor (TNF)-α	+	Induces the expression of adhesion molecules and MHC molecules, the activation of Ag-presenting cells, the production of other inflammatory cytokines, such as interleukin (IL)-1β and IL-6, and induces phagocytosis	(15)
IL-21	+/−	Induces B cell differentiation and Ig production. Stimulates cytotoxicity of natural killer cells. Enhances phagocytosis by macrophages	(59)

**Figure 2:**
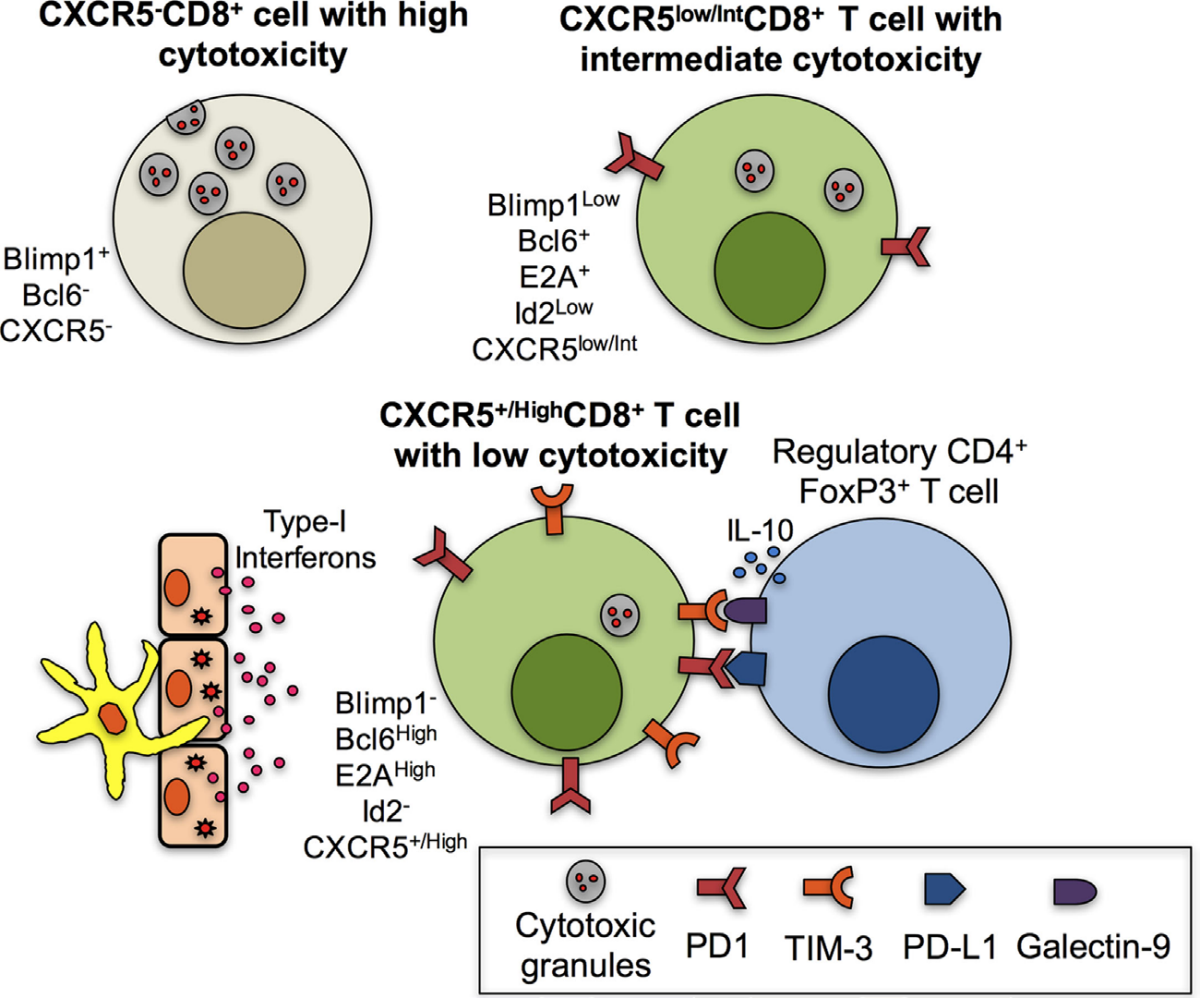
Mechanisms associated with the low cytotoxic function of CXCR5^+^CD8^+^ T cells. CXCR5^−^CD8^+^ T cells are characterized by high levels of cytotoxic granules due to, among other factors, the expression of Blimp1 and the absence of B cell lymphoma 6 protein (Bcl6). CXCR5^low/int^CD8^+^ T cells have decreased cytotoxic functions due to the expression of Bcl6, E2A, and low Blimp1 and Id2 and possibly to the expression of PD-1. However, fully differentiated CXCR5^+/High^CD8^+^ T cells have high expression of Bcl6 and E2A and lack of Blimp1 and Id2. In addition, in the context of chronic infection in the lymphoid follicle and antigenic persistence, the paracrine or endocrine production of type-I interferons (IFNs) by antigen-presenting cells or epithelial cells contributes to the high expression of programmed death (PD)-1 and possibly T-cell immunoglobulin and mucin-domain containing (TIM)-3, which bind to their ligands PD-L1 and galectin-9, respectively, expressed in regulatory CD4^+^ T cells; the IL-10 production by regulatory T cells also contributes to the low cytotoxic abilities of CXCR5^+/High^CD8^+^ T cells.

A number of factors may explain the previous divergent observations. First, it is possible that CXCR5^+^CD8^+^ T cells that possess cytotoxic effector functions constitute non-fully differentiated follicular CD8^+^ T cells that have not reach their phenotypic and functional profile (Figure 1B). Second, a great heterogeneity could exist within the population of CXCR5^+^CD8^+^ T cells in lymphoid follicles, GCs and in circulation (5), according to the infectious or inflammatory scenario evaluated, that could up- and downregulate chemokine receptors, activation markers or effector functions. Third, the expression of inhibitory receptors that confer an exhaustion state, such as PD-1, could, at least in part, play a role in the modulation of the proliferation, differentiation and functionality of CXCR5^+^CD8^+^ T cells. This is supported by the increase in the frequency of CXCR5^+^CD8^+^ T and CXCR5^−^CD8^+^ T cells after the blockade of PD-1–PD-L1 pathway after transfer of CXCR5^+^CD8^+^ T cells to receptor mice (16). This treatment also improved the lytic and non-lytic functions of Ag-specific CXCR5^+^CD8^+^ T and CXCR5^−^CD8^+^ T cells (69, 70). Fourth, a fraction of SIV-specific CD8^+^ T cells inside the lymphoid follicle was found in direct contact with the forkhead box P3 (FoxP3)-positive cells (the classical transcription factor of regulatory cells), and when the percentage of these cells in contact was high, there was a tendency to observe increased SIV levels (18). Thus, follicular regulatory cells might inhibit CXCR5^+^CD8^+^ T cells *in vivo* and this effect could impair the viral control. Fifth, the uncontrolled production of type-I IFNs during chronic infections drives T cell exhaustion (71) and inhibits T_FH_ cells differentiation (72). In the context of chronic viral infections (a model extensively used for the study of CXCR5^+^CD8^+^ T cells), these cytokines might also affect CXCR5^+^CD8^+^ T cells differentiation, increase their exhaustion state, and decrease even more their cytotoxic activity (43). The upregulation of cytolytic functions and degranulation of CXCR5^+^CD8^+^ T cells might occur only after Ag-specific or polyclonal stimulation (45). Sixth, the amount of Ag might be different among different lymph nodes compartments depending on the viral infection. For example, LCMV strains that replicate predominantly outside the follicle would elicit higher cytotoxic responses by extra-follicular CD8^+^ T cells rather than those in the follicle. On the other hand, strains such as DOCILE LCMV elicits a potent cytotoxic response by CXCR5^+^CD8^+^ T cells due to its replication in T_FH_ cells (17). Finally, the magnitude of the expression of Id2, E2A, and other transcription factors modulates the cytotoxic ability of CXCR5^+^CD8^+^ T cells, directs them to follicular or extra-follicular areas, and dictates the fate of this subset (15). In summary, the evidence suggests an important role of CXCR5^+^CD8^+^ T cells in the control of viral infections, even though their differentiation program and the follicle microenvironment regulate some cytotoxic functions (Figure 2). Therefore, the mechanisms through which this population (and possible subtypes) plays this role are poorly understood and require further evaluation.

As shown in Table 3, CXCR5^+^CD8^+^ T cells may also produce various cytokines. During acute LCMV infection, CD44^+^CXCR5^+^CD8^+^ cells expressed low IFN-γ (17), while during chronic infection by the same virus and peptide *ex vivo* stimulation, LCMV-specific murine CD44^+^CXCR5^+^CD8^+^ cells had higher production of IFN-γ and tumor necrosis factor (TNF)-α in comparison with the CXCR5^−^ counterparts. Human CXCR5^+^CD8^+^ cells also express intracellular IFN-γ, TNF-α, and MIP-1β during HIV infection (15, 70). Together, these cytokines support the activation of APCs, promoting the polarization of naïve CD4^+^ T cells to the Th1 profile of CD4^+^ T cells, sustaining the activation of CD8^+^ T cells and ultimately contributing to the control of intracellular pathogens and malignancies.

Some evidence supports a B cell-stimulating function of CXCR5^+^CD8^+^ T cells and their role in the maintenance of follicles and GCs architecture. A study reported an increase in the survival of B cells and in the production of IgG when they were co-cultured with CXCR5^+^CD8^+^ cells, effect that was not observed with CXCR5^−^CD8^+^ cells (5). At least in part, this effect is due to the production of IL-21, a cytokine typically produced by T_FH_ cells which, among other functions, promotes the differentiation of B cells and antibody production (73). Interestingly, murine CD8^+^ T cells can also produce IL-21 after IL-6 stimulation. This conditioned medium induced the secretion of IgG1 by wild-type B cells but not by those with IL-21 receptor deficiency. *In vivo*, these IL-21-producing CD8^+^ T cells were in close contact with follicular and GC B cells in the lung after influenza infection and stimulated the production of protective IgG (59). Thus, although in other study, IL-21-producing CXCR5^+^CD8^+^ T cells were not detected (17), probably in the proper conditions of differentiation (TGF-β plus IL-12 or IL-23 in humans and IL-6 plus IL-21 in mice), CD8^+^ T cells bearing the specific cytokine receptors could generate IL-21-producing CXCR5^+^CD8^+^ T cells and play a role, similar to that of T_FH_ cells, in the stimulation of B cells antibody response. Strikingly, the addition of CXCR5^+^CD8^+^ T cells to co-cultures of B cells and CXCR5^+^CD4^+^ T cells increased the levels of secreted Ig, indicating that CXCR5^+^CD8^+^ T cells indirectly activate B cells *via* stimulation of CD4^+^ T cells or directly through cytokine secretion but not by cell–cell contact (74). Other factors that could be produced by CXCR5^+^CD8^+^ T cells are the B-cell-activating factor of the TNF family (BAFF) and a proliferation-inducing ligand (APRIL), which are produced by several cell populations, such as DCs and neutrophils, promoting the survival and differentiation of B cells (75). Accounting for an important role in the origin and maintenance of GCs, the depletion of murine total CD8^+^ T cells led to the disintegration of follicles, lower transcription of lymphotoxin-β and the _F_DCs marker CD21L (both critical for GCs generation), and loss of antibody production by B cells (12). Notably, the expression of CD40L, CD70, OX40, and ICOS by CXCR5^+^CD8^+^ T cells (5, 16, 17) suggests a receptor-mediated cooperative interaction between this population and B cells. Together, these data indicate that CXCR5^+^CD8^+^ T cells might resemble T_FH_ cells in the ability to stimulate B cells, to promote Ag-specific antibody responses, and to generate and maintain follicles and GCs.

Ultimately, other functions of CXCR5^+^CD8^+^ T cells might include, but not limit to, the constitution and repopulation of the pool of memory CD8^+^ T cells (16) and the regulation of immune responses inside the lymphoid follicle (76). In this regard, a population of follicular regulatory CD8^+^ T cells has been recently described. Similar to the classic regulatory CD8^+^ T cells, this population has immune-suppressive functions in a Qa-1-restricted manner of action (77, 78). Qa-1 is the mouse homolog of the human class Ib MHC HLA-E, expressed by DCs, B cells, and CD4^+^ T cells, that interacts with the TCR of CD8^+^ T cells. As a mode of action, CD4^+^ T cells expressing Qa-1 that bear self-peptides, such as the heat shock protein 60, interact with the TCR of CD8^+^ T cells, inducing their suppressive functions. On the other hand, Qa-1 bearing foreign peptides in DCs binds to NKG2A in CD8^+^ T cells, inhibiting their cytolytic functions (79). In contrast to follicular regulatory CD4^+^ T cells, which express the transcription factor Fox P3, follicular regulatory CD8^+^ T cells do not have a defined transcriptional program, although Helios and STAT5 pathways are required for their survival and to prevent terminal differentiation, suggesting a divergent differentiation pathway in comparison with non-regulatory follicular CD8^+^ T cells (80). Follicular regulatory CD8^+^ T cells and non-regulatory follicular CD8^+^ T cells share the expression of CD44 and CXCR5, while the expression of the NKG2A receptor, CD122 (the IL-2 receptor β chain, part of the IL-15 receptor), ICOSL, galectin-9, and the murine class I MHC receptor Ly49 (none of them expressed on non-regulatory follicular CD8^+^ T cells) is limited to the regulatory subset. By contrast, PD-1 is mainly expressed by non-regulatory follicular CD8^+^ T cells (76, 81, 82). Consequently, with the expression of CD122, the activity of this subset is boosted by IL-15. Of note, studies that directly compare the phenotypic differences between follicular regulatory and non-regulatory CD8^+^ T cells are required.

**Figure 3:**
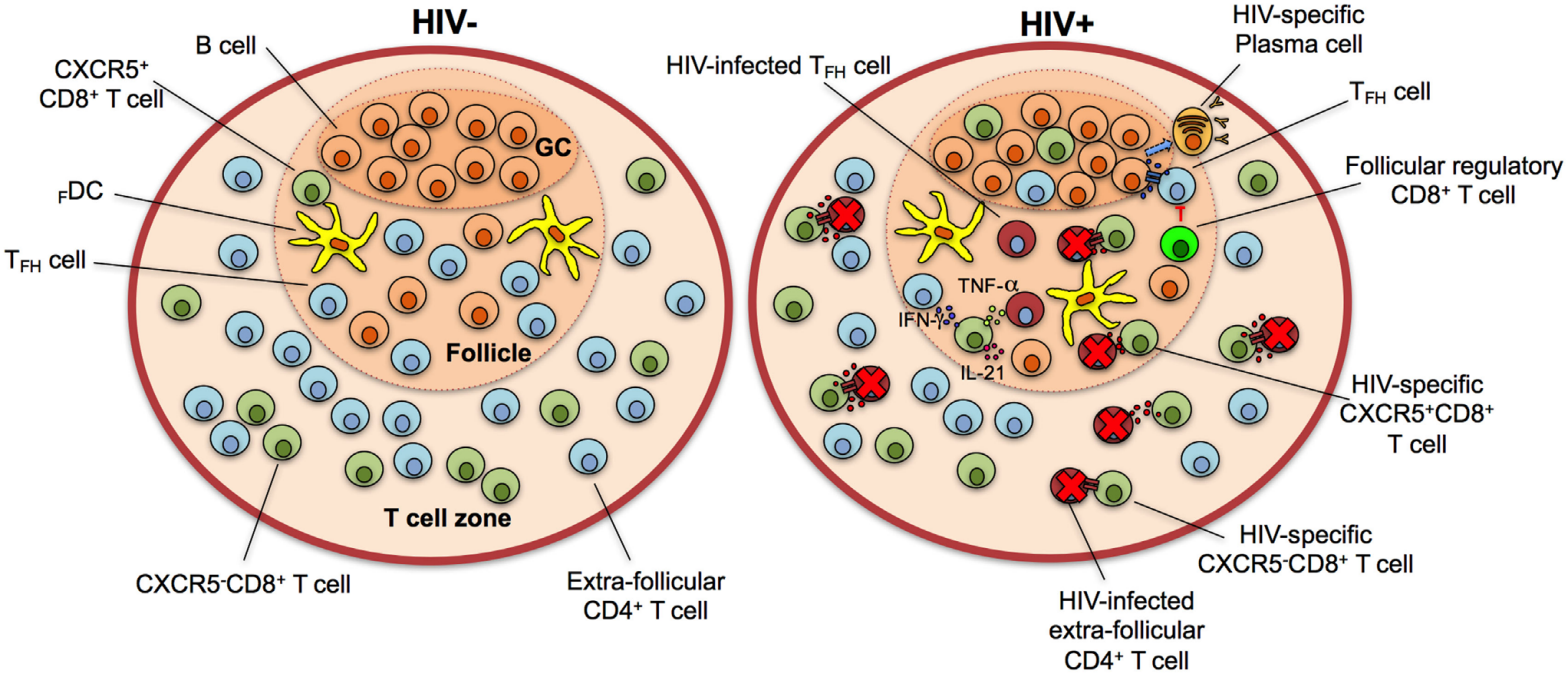
CXCR5^+^CD8^+^ T cells control human immunodeficiency virus (HIV) infection in the lymphoid follicle. In HIV-negative individuals, the majority of CD8^+^ T cells localize in the T cell zone, with a minor fraction present in the lymphoid follicle. During HIV infection, the population of CXCR5^+^CD8^+^ T cells increases in lymphoid follicles and GCs and Ag-specific cytotoxic cells eliminate infected follicular helper T cells (T_FH_) cells through the release of cytotoxic granules and/or receptor-mediated apoptosis. They also secrete interferon (IFN)-γ, tumor necrosis factor (TNF)-α, and interleukin (IL)-21 to stimulate T_FH_ and B cells and contribute to an antiviral state. Follicular regulatory cytotoxic cells possibly inhibit T_FH_ cells, which in turn provide stimuli to B cells for their differentiation to HIV-specific plasma cells with production of high-affinity, isotype class-switched antibodies. Note that the frequency of HIV-infected but not eliminated cells is higher in the follicle in comparison with extra-follicular areas. GC, germinal center.

The role of follicular regulatory CD8^+^ T cells in the control of autoimmune syndromes was evidenced in mice bearing a single mutation in Qa-1 protein, which developed lupus-like disease due to the absence of suppression on T_FH_ cells, which in turn stimulated the production of autoantibodies by B cells (76). Similar findings were obtained in B6-Y*aa* mice, characterized by increased frequencies of T_FH_ and GCs cells and lupus-like syndrome, where a defect in follicular regulatory CD8^+^ T cells is probably the cause of the disorder (81). Recently, follicular regulatory CD8^+^ T cells were found to have a role in HIV infection. In *ex vivo* HIV-infected human tonsils, CD3^+^CD8^+^CXCR5^hi^CD44^hi^ regulatory cells expressed high levels of TIM-3, CD122, CD215 (IL-15 receptor α chain), and IL-10 and low perforin, comprising more than 90% of CXCR5^+^CR7^−^ cells. The frequency of this population also increased during SIV chronic infection. Strikingly, follicular regulatory CD8^+^ T cells inhibited T_FH_ cells function, affecting IgG production by B cells, and induced their apoptosis in a TIM-3 and HLA-E-dependent manner, respectively (Figure 3). This suppression of T_FH_ cells activation apparently was responsible of a slight decrease in HIV infection of these cells, although at expense of the humoral response (82). Thus, follicular regulatory CD8^+^ T cells could have a myriad of potential functions and roles in autoimmune and infectious diseases. Maybe, the most important and unanswered questions are their relation with the “classical” CXCR5^+^CD8^+^ T cell subset and if their activity is beneficial or not, depending on the context.

## CXCR5^+^CD8^+^ T Cells in Disease

### Malignancies

CD8^+^ T cells located in follicular areas are important in the control and associated with the prognosis in a number of cancer types, such as B cell, follicular, and Hodgkin lymphomas (83–85). These types of tumors require a proper microenvironment to fully develop (86). Furthermore, lymphoid follicles and the partial absence of CD8^+^ T cells provide the conditions for tumor growth and immune evasion. In agreement with a potential role of CXCR5^+^CD8^+^ T cells in the control of malignancies inside the lymphoid follicle, B cell lymphoma-bearing mice showed increased frequencies of CXCR5^+^CD44^+^CD8^+^ cells; in addition, B cells infected with murid herpes virus 4 (associated with B cell transformation) were also eliminated by this subset *in vivo* (17). Thus, CXCR5^+^CD8^+^ T could be relevant for the immune surveillance and control of cancer in the lymphoid follicles.

### SIV and HIV Infections

We previously discussed the control of LCMV infection exerted by CXCR5^+^CD8^+^ T cells. However, SIV and HIV infections and acquired immunodeficiency syndrome (AIDS) constitute excellent models for the study of CXCR5^+^CD8^+^ T cells due to the high compromise of follicle cells in this disease.

Human immunodeficiency virus has tropism for CD4^+^ T cells, which are progressively depleted during primary infection (87). Activated cells are preferentially eliminated within the first days, but during the subsequent weeks, almost all immune cells are affected by the uncontrolled immune activation phenomenon. As resting memory CD4^+^ T cells with integrated viral DNA persist throughout the time, the absence of replication in these cells limits HIV eradication by the anti-retroviral therapy; therefore, a cure is not achieved (88). These infected but not replicating cells constitute HIV reservoirs and locate preferentially in secondary lymphoid organs (89) due to the proper survival conditions that they provide to resting memory cells (90). These reservoirs explain the rapid viral rebound observed after suspension of the treatment in patients with previous sustained viral control (91). In addition to memory T cells, T_FH_ cells are also latently infected; in fact, this compartment contains more infected cells than any other CD4^+^ T cell subset (92). _F_DCs also adhere HIV virions through their highly expressed complement receptors CR1 and CR2 or the DC-specific, ICAM-3 grabbing non-integrin (DC-SIGN) (93, 94) and retain infectious virus within non-degradative cycling endosomes, allowing the infection of uninfected CD4^+^ T cells (95). Thus, the persistence of HIV virions close to a large pool of susceptible cells, in addition to the low frequency of cytotoxic cells, makes the lymphoid follicle an ideal environment for HIV maintenance.

CD8^+^ T cells are critical for the control of HIV infection. This has been demonstrated by the decrease of viral load with the appearance of virus-specific CD8^+^ T cells (96, 97), its increase after their depletion (98) and the higher frequency and functionality of CD8^+^ T cells in HIV-infected non-progressors individuals (99, 100). CD8^+^ T cells are largely confined to secondary lymphoid organs during natural HIV infection and migrate to these locations after autologous adoptive transfer (101–103). There, they preferentially locate in the T cell zones (extra-follicular zones), correlating with high levels of the antiviral proteins α-defensins, RANTES, and MIP-1α, thus efficiently controlling the HIV replication (104, 105). Indeed, after depletion of CD8^+^ T cells, the frequency of infected cells in extra-follicular areas increases at similar levels to that of follicular areas (8). Together, these data support the immune privilege of the lymphoid follicle—possibly to prevent the alteration in B cell antibody responses—which is exploited for some viruses, such as HIV and human herpes virus 8, to establish their reservoirs (104, 106).

Despite the preferential extra-follicular location of CD8^+^ T cells during HIV infection, recent evidence demonstrates a role of CXCR5^+^CD8^+^ T cells in SIV and HIV infections. The first descriptions of the presence of follicular CD8^+^ T cells came from studies in lymph nodes from HIV-infected individuals, where the increase in the frequency of this cell type was associated with HIV-induced follicular hyperplasia (10, 107, 108). SIV- and HIV-specific CD8^+^ cells were also detectable in lymphoid follicles, splenic white pulp, and GCs (109, 110), promoting the elimination of infected CD4^+^ T cells (111). Notably, HIV replication in non-treated individuals triggered Ag-specific CD8^+^ T cell responses in secondary lymphoid organs, while during anti-retroviral therapy this response was not readily detectable, suggesting that their expansion is Ag-induced (109). Possibly these CD8^+^ T cells in secondary lymphoid organs constitute a memory population and are responsible of an expansion of Ag-specific responses in blood after anti-retroviral treatment interruption and viral rebound (102). Thus, HIV infection induces the migration of CD8^+^ T cells to secondary lymphoid organs and the anti-retroviral therapy—with a consequent decrease in viral load—at least in part modulates their frequency and function.

Accordingly, the frequency of CXCR5^+^CD8^+^ T cells in lymph nodes, but not that of CXCR5^−^CD8^+^ T cells, is higher in HIV-infected patients than in healthy controls, and they are in close proximity to HIV RNA^+^ cells (15, 17). In HIV-infected patients, the frequency of effector CXCR5^+^CD8^+^ T cells is higher than their naïve counterparts, indicating an increase of the migration to the follicle of this population during primary infection (17). Nevertheless, apparently HIV promotes and maintains the CXCR5^+^CD8^+^ T cells response indirectly through the induction of local inflammation and immune activation (such as the production of CXCL13 and myeloperoxidase) but not through the direct effect of antigenic stimulation (70). Interestingly, the frequency of SIV-specific CXCR5^+^CD8^+^ T cells inversely correlated with that of infected T_FH_ cells and plasma viral load, highlighting a role of CXCR5^+^CD8^+^ T cells in the control of local and systemic lentiviral infections (45).

Similar to previously discussed effector mechanisms of CXCR5^+^CD8^+^ T cells, in HIV infection, this subset has the ability to produce MIP-1β, IFN-γ, and/or TNF-α, but not simultaneously, a feature explained at least in part by the inhibitory effect of the PD-1–PD-L1 pathway (70). CXCR5^+^CD8^+^ T cells isolated from SIV-infected rhesus macaques or HIV-infected individuals also express cytotoxic molecules and efficiently eliminate infected cells *in vitro* (45, 70). Importantly, CXCR5^+^CD8^+^ T cells from HIV viremic non-treated patients co-express higher granzyme B and perforin levels than treated patients (70) supporting the hypothesis of the requirement of the antigenic exposure and inflammatory microenvironment for the maintenance of CXCR5^+^CD8^+^ T cells response during HIV infection. The exhaustion state is, however, the counterpart of this persistent stimulation.

CXCR5^+^CD8^+^ T cells are also present in peripheral blood during HIV chronic infection, express cytokines and cytotoxic molecules, and represent almost the 20% of circulating HIV-specific CD8^+^ T cells (15). Strikingly, the frequency of circulating HIV-specific CXCR5^+^CD8^+^ T cells inversely correlated with HIV serum viral load, suggesting its potential as a correlate of protection (15, 17).

Notably, the control of lentivirus infections by CXCR5^+^CD8^+^ T cells in the lymphoid follicle is not complete. Although inverse correlations have been found between SIV-specific CXCR5^+^CD8^+^ T cells and follicle viral load, these correlations are stronger when CD8^+^ T cells in extra-follicular areas are analyzed (18). SIV-producing cells are also persistently detectable in lymphoid follicles throughout the disease stages and their levels are higher in comparison with those in extra-follicular zones (4). Moreover, after CD8^+^ cells depletion, the levels of SIV-producing cells is preferentially augmented in extra-follicular areas, demonstrating a more relevant and effective role of extra-follicular CD8^+^ T cells in viral control (8, 18). Thus, SIV and HIV infections apparently induce a higher presence of CXCR5^+^CD8^+^ T cells in the lymphoid follicle, but they cannot eliminate SIV- or HIV-infected cells as efficiently as CD8^+^ T cells outside the follicle (Figure 3). Besides the intrinsic biology of CXCR5^+^CD8^+^ T cells, the antigenic persistence, inflammatory environment, and clinical scenario may account for the dynamics of this population in SIV and HIV infections. In addition, the regulation of follicular cytotoxic effects (that could affect the B cells antibody production) might explain the heterogeneous CXCR5^+^CD8^+^ T cells response in health and disease.

## Remaining Questions and Possible Strategies to Boost the Elimination of HIV Reservoirs in Lymphoid Follicles

An increasing interest in CXCR5^+^CD8^+^ T cells has revealed some striking characteristics of this cell subset; however, more questions than answers remain yet. For example, little is known about the cytokines or soluble factors that induce the differentiation of CD8^+^ T cells to the CXCR5^+^CD8^+^ T cell profile that could be helpful for *in vitro* studies or as a therapeutic strategy to increase the frequency and location of this cell subset in the lymphoid follicles. The combination of TGF-β plus IL-12 or IL-23 is proposed for this purpose.

The transcriptional profile of CXCR5^+^CD8^+^ T cells promotes their entry into follicles but modulates their cytotoxic ability. An important aspect to be addressed is how to boost the entry into follicles of this population without affecting the follicle homeostasis and the pool of extra-follicular CD8^+^ T cells. Defining the specific transcription factors that downregulate cytotoxic molecules and designing new strategies to modulate them (e.g., siRNA or miRNA) deserves further exploration. In addition, the adoptive transfer of autologous HIV-specific CXCR5^+^CD8^+^ T cells (e.g., expanded *ex vivo* through treatment with differentiation factors), in conjunction with potentiators such as bispecific antibodies is another approach to increase the levels of CXCR5^+^CD8^+^ T cells that more efficiently eliminate virus-producing cells (70). Moreover, the stimulation of CXCR5^+^CD8^+^ T cells with IL-27 could potentiate the cytotoxic properties of this subset, along with the blockade of the suppression exerted by T regulatory cells, as was shown in murine metastatic neuroblastoma (112). The expression of PD-1 in CXCR5^+^CD8^+^ T cells suggests a state of exhaustion of this population that could decrease their lytic and non-lytic functions or even restrict their entry to lymphoid follicles. Thus, the blockade of PD-1–PD-L1 pathway is another feasible strategy to promote the CXCR5^+^CD8^+^ T cells function (15, 70), as it has been proved in anti-tumor CD8^+^ T cells (113, 114).

Notably, the HIV reservoirs in lymphoid follicles are characterized by latently infected resting cells where the virus is not replicating and anti-retroviral therapy cannot efficiently eliminate these reservoirs because of their focused capacity to eliminate replicating virus (115). Likewise, CD8^+^ T cells require antigenic pre-stimulation to clear reactivated virus (116). Thus, the combination of the aforementioned strategies of CXCR5^+^CD8^+^ T stimulation with molecules that reverse the latency state of HIV (such as histone deacetylase or methyltransferase inhibitors), plus antigenic pre-stimulation, would be required for eradication of HIV reservoirs by these cytotoxic cells.

Taking into account that CXCR5^+^CD8^+^ T cells do not possess the same cytotoxic function than classic CD8^+^ T cells, it would be interesting to determine the cytotoxic or non-cytotoxic mechanisms that this population employ for the HIV control during natural infection in humans and their role in different contexts of the infection, such as acute or chronic phases, immune hyper-activation states, or in elite controllers and progressors. These mechanisms could be also boosted through therapeutic or vaccine strategies. Likewise, the beneficial or harmful role of follicular regulatory CD8^+^ T cells in HIV infection deserves to be evaluated. As current data demonstrate a dampened humoral response induced by this cell subtype, their function should also be taken into account in the design of HIV vaccines and the induction of the non-regulatory profile of CXCR5^+^CD8^+^ T cells should be explored. Finally, the role of CXCR5^+^CD8^+^ T cells in the response to the anti-retroviral therapy and their phenotypic or functional modifications during viral, immune, or clinical failure could provide new insights for improving anti-HIV treatment. In this regard, based on the potential antiviral effect of CXCR5^+^CD8^+^ T cells, we anticipate a role of this population in maintaining the response to anti-retroviral therapy, preventing treatment failure and promoting immune reconstitution after exerting viral control.

## Author Contributions

FP-C wrote the manuscript and designed the figures; NT and MR critically edited the manuscript.

## Conflict of Interest Statement

The authors declare that the research was conducted in the absence of any commercial or financial relationships that could be construed as a potential conflict of interest.
